# Supplementary figures and images for: Enhanced production of l-fuculose by Escherichia coli engineered via genome-scale metabolic modeling
Source: PLoS One. 2026 May 14;21(5):e0349374. doi: 10.1371/journal.pone.0349374 (PMC13175319; doi:10.1371/journal.pone.0349374)

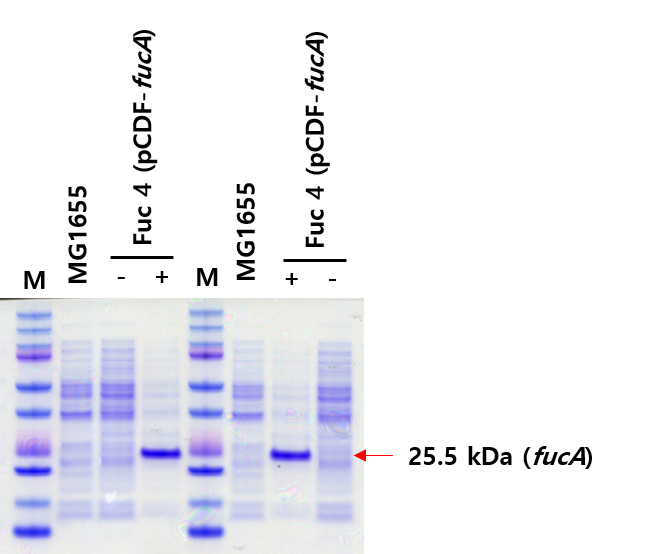

Supplement: S1 Fig — Protein expression was induced with IPTG, and whole-cell lysates were analyzed by SDS-PAGE. A prominent band corresponding to the expected molecular weight of fucA was observed in the IPTG-induced sample, whereas this band was absent or significantly weaker in the non-induced control, confirming successful overexpression of fucA. (PNG) [file pone.0349374.s004.png]
