# Supplementary material for: Enhanced production of l-fuculose by Escherichia coli engineered via genome-scale metabolic modeling
Source: PLoS One. 2026 May 14;21(5):e0349374. doi: 10.1371/journal.pone.0349374 (PMC13175319; doi:10.1371/journal.pone.0349374)
Supplement: Attachment — Submitted filename: 260331_Response to Reviewer Comments.docx [file pone.0349374.s006.docx]

**Response to Reviewers’ Comments**

Dear Editor and Reviewer,

We sincerely thank the editor and reviewer for their careful evaluation and constructive comments. We have revised the manuscript accordingly and believe that these changes have significantly improved the clarity and quality of our work. Below, we provide a point-by-point response to all comments.

**<Editorial Comment>**

**[Comments]**

**Comment 1.** Please provide growth curves and microscopic images (e.g., confocal microscopy) for all generated strains alongside the appropriate control samples.

***Response:*** We thank the editor for this valuable suggestion. Growth curves for all engineered strains have been included in the revised manuscript (Fig. 2 and 3).

Regarding microscopic analysis, while such data may provide additional insights into cellular morphology, the genetic modifications introduced in this study were not expected to induce significant morphological changes. As the primary focus of this study was on metabolic flux redistribution and L-fuculose production, and growth characteristics were already evaluated through growth curves, we believe that the current dataset sufficiently supports our conclusions. Microscopic analysis may be considered in future studies.

**[Revision: Figs. 2 and 3]**

**Comment 2.** How were the knockout (KO) and knock-in (KI) strains verified? Any PCR validation data must be included. Furthermore, evidence supporting the overexpression of *fucA* should also be provided.

***Response:*** We thank the editor for this important comment. The knockout strains were verified by PCR during strain construction, as described in the Methods section (Construction of mutants). In addition, evidence supporting the overexpression of fucA has been added to the revised manuscript. As shown in Fig. S1, SDS-PAGE analysis revealed a prominent band at the expected molecular weight of FucA following IPTG induction, confirming successful overexpression.

**[Revision: Fig. S1]**

**Comment 3.** The discussion section is currently unsatisfactory and requires more careful revision. Overstatements should be strictly avoided and conclusions must be supported by the results presented in this study.

***Response:*** We thank the editor for this valuable comment. The Discussion section has been carefully revised to avoid overstatements and to ensure that all conclusions are strictly supported by the experimental results. The language has been moderated throughout to better reflect the scope and limitations of the study.

**[Revision: Lines 249-254]**

Compared to *Saccharomyces cerevisiae* (used in a previous study), which produced only 1.55 mg/L of l-fuculose [1], the Fuc 4 strain achieved more than a 30-fold improvement, suggesting the potential of *E. coli* as a host for L-fuculose biosynthesis. Although aldA deletion was intended to block the conversion of LAD to lactate, lactate levels were not directly quantified in this study. Therefore, the contribution of aldA deletion to reducing lactate formation remains to be further validated.

**<Reviewer 1>**

**[Comments]**

**Comment 1.** It is suggested to show the dynamic production of L-fuculose during the fermentation.

***Response:*** We thank the reviewer for this helpful suggestion. The time-dependent production of L-fuculose during fermentation has now been incorporated into the revised manuscript and is presented in Fig. 3. Corresponding descriptions have been added in the Results section.

**[Revision: Lines 203-207; Fig. 3]**

**Fig 3. L-fuculose production during fermentation and confirmation by mass spectrometry.** (A) L-Fuculose production by engineered *E. coli* strains (Fuc 1–4). Data are presented as mean ± standard deviation. (B) Mass spectrum of L-fuculose produced by the engineered strain. (C) Mass spectrum of the analytical L-fuculose standard.

LAD was rapidly depleted within the first 12 h, whereas 1,2-PDO began to accumulate from 12 h, with its concentration increasing gradually until the end of fermentation. L-fuculose production was detected after 12 h and gradually increased throughout fermentation, reaching 38.92 ± 5.19 mg/L (Fig 3A). GC/MS analysis of the culture supernatant confirmed the presence of L-fuculose (Fig 3B, C).

**Comment 2.** Considerable acetate was accumulated. Discussion is required.

***Response:*** We thank the reviewer for this important comment. A discussion on acetate accumulation has been added to the revised manuscript. We now describe that acetate accumulation likely reflects overflow metabolism in *E. coli* under high carbon flux conditions and may reduce carbon availability for L-fuculose biosynthesis.

**[Revision: Lines 258-261]**

In addition, the accumulation of acetate observed during fermentation likely reflects overflow metabolism in *E. coli* under high carbon flux conditions, which can divert carbon away from the desired product and reduce L-fuculose production efficiency [18].

**Comment 3.** AldA was deleted to block conversion of LAD to lactate. Was accumulation of lactate detected before and after aldA deletion?

***Response:*** We thank the reviewer for this insightful comment. Although aldA deletion was intended to block the conversion of LAD to lactate, lactate levels were not directly quantified in this study. This limitation has been acknowledged in the revised manuscript, and future studies will be required to further evaluate the contribution of aldA deletion to lactate reduction.

**[Revision: Lines 251-254]**

Although aldA deletion was intended to block the conversion of LAD to lactate, lactate levels were not directly quantified in this study. Therefore, the contribution of aldA deletion to reducing lactate formation remains to be further validated.

**Comment 4.** fucA::T7, what’s the meaning of T7 here? Which promoter was applied to express fucA?

***Response:*** We thank the reviewer for this comment. The description has been clarified in the revised manuscript to indicate that fucA was expressed under the control of the T7 promoter.

**[Revision: Lines 196-200]**

In addition, *fucA* was overexpressed under the control of the T7 promoter to catalyze the aldol condensation of LAD and DHAP to L-fuculose-1-phosphate. Overexpression of *fucA* was confirmed by SDS-PAGE analysis following IPTG induction (Fig. S1), showing a distinct band at the expected molecular weight. The resulting strain, designated Fuc 1 (Δ*fucI* Δ*fucK*, *fucA* overexpression), was cultivated in glucose–LAD medium for 96 h.

**Comment 5.** It is suggested to perform the fermentation in a bioreactor to further indicate the significance of this study.

***Response:*** We thank the reviewer for this valuable suggestion. While the present study was conducted in shake-flask cultures, we have added a statement in the Discussion section indicating that future studies involving bioreactor-scale fermentation will be important to evaluate scalability and industrial applicability.

**[Revision: Lines 268-271]**

Although this study was performed in shake-flask cultures, future studies involving bioreactor-scale fermentation will be important to validate the scalability and industrial relevance of the proposed strategy.
